# Fly Poison

**Published:** 1853-08

**Authors:** 


From the Southern Medical and Surgical Journal.

*Fly Poison.*—The danger of using the article commonly called Cobalt or Fly-Stone, the activity of which depends upon the arsenic it contains, renders it desirable to get an innocent substitute for it. The Swiss, it is said, are in the habit of using, very effectually, a strong decoction of quassia amara well sweetened with molasses. The flies being fond of it seek it with avidity and are very promptly destroyed.

It is said that an infusion or decoction of our poke root or the juice of its berries, made sweet with molasses, will very effectually destroy cockroaches. We have tried strewing the floor with cucumber peelings, and found it better than anything we ever used for getting rid of these filthy insects.

